# The disease sites of female genital cancers of *BRCA1*/*2*-associated hereditary breast and ovarian cancer: a retrospective study

**DOI:** 10.1186/s12957-021-02151-3

**Published:** 2021-02-02

**Authors:** Takashi Mitamura, Masayuki Sekine, Masami Arai, Yuka Shibata, Momoko Kato, Shiro Yokoyama, Hiroko Yamashita, Hidemichi Watari, Ichiro Yabe, Hiroyuki Nomura, Takayuki Enomoto, Seigo Nakamura, and the Registration Committee of the Japanese HBOC consortium

**Affiliations:** 1 Department of Obstetrics and Gynecology, Faculty of Medicine and Graduate School of Medicine, Hokkaido University, Kita 14, Nishi 5, Kita-ku, Sapporo, Hokkaido 060-8648 Japan; 2 Division of Clinical Genetics, Hokkaido University Hospital, Sapporo, Japan; 3 Department of Obstetrics and Gynecology, Niigata University Graduate School of Medical and Dental Sciences, Niigata, Japan; 4 Clinical Genetics, Juntendo University, Graduate School of Medicine, Tokyo, Japan; 5 Division of Breast Surgical Oncology, Department of Surgery, Showa University School of Medicine, Tokyo, Japan; 6 Department of Breast Surgery, Hokkaido University Hospital, Sapporo, Japan; 7 Department of Obstetrics and Gynecology, School of Medicine, Fujita Health University, Toyoake, Japan

**Keywords:** Hereditary breast and ovarian cancer, Ovarian cancer, Fallopian tube cancer, Peritoneal cancer, *BRCA1*, *BRCA2*

## Abstract

Disease sites of female genital tract cancers of *BRCA1*/*2*-associated hereditary breast and ovarian cancer (HBOC) are less understood than non-hereditary cancers. We aimed to elucidate the disease site distribution of genital cancers in women with the germline *BRCA1* and *BRCA2* pathogenic variants *(BRCA1*+ and *BRCA2*+) of HBOC. For the primary disease site, the proportion of fallopian tube and peritoneal cancer was significantly higher in *BRCA2*+ (40.5%) compared with *BRCA1*+ (15.4%) and *BRCA−* (no pathogenic variant, 12.8%). For the metastatic site, the proportion of peritoneal dissemination was significantly higher in *BRCA1*+ (71.9%) than *BRCA−* (55.1%) and not different from *BRCA2*+ (71.4%). With one of the most extensive patients, this study supported the previous reports showing that the pathogenic variants of *BRCA1*/*2* were involved in the female genitalia’s disease sites.

## Background

Disease sites of female genital tract cancers of hereditary breast and ovarian cancer (HBOC) are less understood than non-hereditary cancers. We aimed to elucidate the disease distribution of ovarian, fallopian tube, and primary peritoneal cancers in women with the germline *BRCA1* and *BRCA2* pathogenic variants (*BRCA1*+ and *BRCA2*+) of HBOC.

## Materials and methods

The ethics review board of the Japanese HBOC Consortium approved the establishment of the database and the future publication of our analysis results on February 18, 2016, to investigate Japanese HBOC patients’ characteristics. The registered subjects were any women who consecutively underwent blood *BRCA1* and *BRCA2* genetic testing and agreed with this study from 2016 to 2018. We carried out the genetic testing of germline *BRCA1* and *BRCA2* in the 80 medical institutions where genetic counseling by certified specialists was available. We accepted all genetic testing purposes, including clinical practice for diagnosing *BRCA1*/*2*-associated HBOC or decision of PARP inhibitors’ indication or translational research. For clinical practice, we usually used the testing criteria which NCCN published during the study period [1]. To collect rough data on a large number of *BRCA1/2*-associated HBOC cancer patients, we did not set any exclusion criteria on age, family history, the modalities and intervals for image testing, and personal history of *BRCA1*/*2*-associated HBOC cancers. To confirm whether *BRCA1/2* variants were pathogenic or not, we used the Myriad Genetic Laboratories database of the latest version at the time of testing in 90.1% of patients. We also carried out the other tests at the facilities of the investigators or other research institutes. We finally checked the latest information on ClinVar (the archival database at the National Center for Biotechnology Information [NCBI], https://www.ncbi.nlm.nih.gov/clinvar/) in 2020 and confirmed there were no critical changes for pathogenicity. We showed the pathogenic variants registered in our database elsewhere [2]. We retrospectively reviewed the data on patients in the *BRCA*− (no pathogenic variant), *BRCA1*+, and *BRCA2*+ groups, and investigated primary disease sites and metastatic sites. We performed all statistical analyses with the JMP® Pro software program, ver. 14.0.0 (SAS Institute, Cary, NC, USA), and compared the proportions of disease sites between each group with Pearson’s *χ*^2^ test or Fisher’s exact test. We considered *P* values of less than 0.05 to be statistically significant.

## Results

The patients’ age (range and median) was 28–83 (49), 41–77 (57), and 12–81 (55) in *BRCA1*+, *BRCA2*+, and *BRCA*−. The proportion of the patients under 40 years of age was significantly lower in *BRCA2*+ (0%) than *BRCA1*+ (7.8%, *P* = 0.01) and *BRCA−* (6.4%, *P* = 0.02). The proportion of the patients with personal breast cancer history was not significantly different between *BRCA*− and *BRCA1*+ (24.1% and 29.0%, *P* = 0.24), and *BRCA*− and *BRCA2*+ (24.1% and 38.1%, *P* = 0.05).

**Fig. 1:**
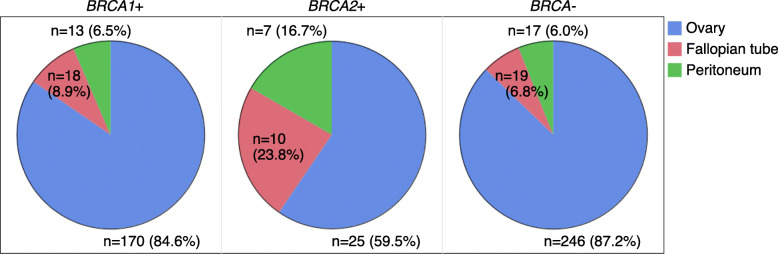
The proportion of primary disease sites of female genital cancers according to *BRCA* variants

**Fig. 2:**
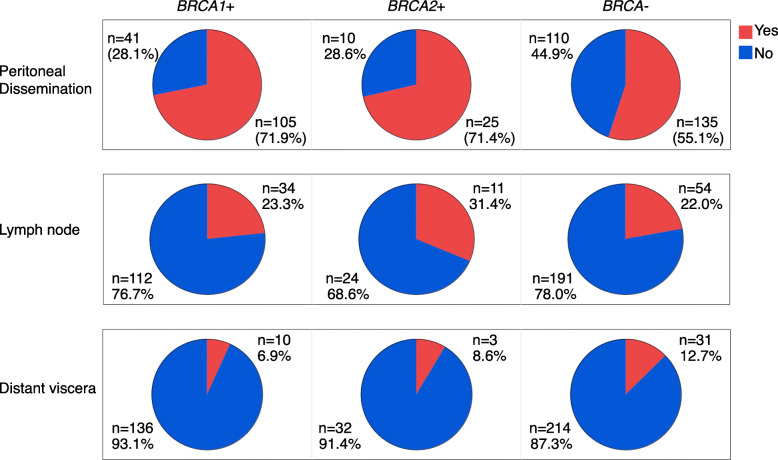
The proportion of metastatic disease sites of female genital cancers according to *BRCA* variants

For primary disease sites, we reviewed the data on 277 patients in *BRCA*−, 190 patients in *BRCA1*+, and 42 patients in *BRCA2*+, respectively (Fig. 1). There were 5 (1.8%) patients in *BRCA*− and 9 (4.7%) in *BRCA1+* patients in whom the clinicians registered two overlapping primary disease sites. The proportion of fallopian tube and peritoneal cancer was significantly higher in *BRCA2*+ (40.5%, *n* = 17) compared with *BRCA1*+ (15.4%, *n* = 31) (*P* < 0.001) and *BRCA*− (12.8%, *n* = 36) (*P*<0.001) and not different between *BRCA1*+ and *BRCA−* (*P* = 0.41). For the metastatic disease site, we reviewed the data on 245, 146, and 35 patients in the *BRCA*−, *BRCA1*+, and *BRCA2*+, whose details of metastasis are available (Fig. 2). The proportion of peritoneal dissemination was significantly higher in *BRCA1*+ (71.9%, *n* = 105) compared with *BRCA−* (55.1%, *n* = 135) (*P* < 0.001), and not significantly different between *BRCA2*+ (71.4%, *n* = 25) and *BRCA−* (*P* = 0.06) and between *BRCA1*+ and *BRCA2*+ (*P* = 0.95). The prevalence of lymph node metastasis was not different between *BRCA1*+ (23.3%, *n* = 34) and *BRCA*− (22.0%, *n* = 54) (*P* = 0.73), between *BRCA2*+ (31.4%, *n* = 11) and *BRCA−* (*P* = 0.22), and between *BRCA1*+ and *BRCA2*+ (*P* = 0.33). The prevalence of distant visceral metastasis was not different between *BRCA1*+ (6.9%, *n* = 10) and *BRCA−* (12.7%, *n* = 31) (*P* = 0.19), between *BRCA2*+ (8.6%, *n* = 3) and *BRCA−* (*P* = 0.59), and between *BRCA1*+ and *BRCA2*+ (*P* = 0.84).

## Discussion

The results of our study suggest that female genital cancer in *BRCA2*+ less frequently originate from the ovary than *BRCA1*+ and *BRCA−*. The disease site distributions by *BRCA* status are similar to those of previous Japanese prospective study [3] where the proportion of fallopian tube and peritoneal cancer was 40% in *BRCA2*+, and 15% in *BRCA1*+, and 14% in *BRCA−*, and our retrospective study with a larger number of *BRCA1*/*2* patients supported these results. Although the proportion is somewhat different, HBOC-related female genital cancer in *BRCA2*+ also less frequently originates from the ovaries than *BRCA1*+ and *BRCA−* in the USA [4] and Australia [5].

Although the frequencies of peritoneal dissemination of *BRCA1*+ and *BRCA2*+ are almost identical, the proportion of peritoneal dissemination was significantly higher when comparing *BRCA1*+ (71.9%) to *BRCA*− (55.1%) but not significantly different between *BRCA2*+ (71.4%) and *BRCA*− (*P* = 0.06). These results might suggest that *BRCA2*+ lacked the power to detect statistical significance due to the small sample size, and we will need further analysis with the larger population. The prevalence of metastasis has been controversial. A previous retrospective study in Italy showed that metastatic sites were the same between *BRCA1*+/*2*+ and *BRCA−* [6]. In contrast, a recent report showed an increased incidence of visceral metastases in Scottish *BRCA1*/*2*-defective ovarian cancer patients [7], and we need a more extensive study to explore whether ethnicity is involved in this difference.

The previous prospective study showed that *BRCA1*+ and *BRCA2*+ consisted of 9.9% and 4.7% of Japanese women with newly diagnosed ovarian cancer [3]. In this study, the proportion of *BRCA1*+ was considerably high (37.3%), and the proportion of *BRCA2*+ was also higher than expected (8.3%). We guess this result suggests selection and institutional bias due to actively conducted genetic testing by medical genetics specialists.

In conclusion, although our study is retrospective, this study, with one of the most extensive patients, supported the previous reports showing that the pathogenic variants of *BRCA1*/*2* were involved in the female genitalia’s disease sites.

## Data Availability

The data that support the findings of this study are available in the previous article [2].

## Abbreviations

HBOC: Hereditary breast and ovarian cancer
*BRCA1*+: Women with the germline *BRCA1* pathogenic variants
*BRCA2*+: Women with the germline *BRCA2* pathogenic variants
*BRCA*−: Women with no pathogenic variant
